# How Can Medical Societies

**Published:** 1889-02

**Authors:** 


### HOW CAN MEDICAL SOCIETIES

### Be Made so Profitable that Their Members shall Feel that to Attend Meetings will Individually Pay Better than to Stay at Home?

To spend his time to secure the greatest profit to himself, is the right of every man. In a sense, such is his duty. In another sense, his profit is greatest when he is serving others. We have often heard doctors say, when asked to attend a certain meeting of a medical society, "I can spend my time more profitably with my medical journals at home."

If this be true, then he should stay at home with his journals.

If medical societies are at their best, those doctors who are familiar with them will know that society meetings are more profitable to them than staying at home reading medical journals. They will know that their professional success will become more assured by attendance upon such meetings. Such is our conviction, at least.

The question now faces us, how shall such meetings be secured?

First, it will be apparent that there can be no stereotyped plan of conducting societies, in different localities, among practitioners of divers experiences, of infinitely varied culture, social and other surroundings. The society in the mountains, the society in the plain, the society in the region of long continued heat, the society in the region of long continued cold, the society of the country, of the village, and of the great city, each of these must work out its own scheme, to meet the pressing needs of its membership, actual or desired.

Second, it is clear that it is of the first importance that the officers be selected not for any imaginary honor which it is desired to confer upon them, but simply and solely for their fitness and willingness to serve the medical profession of a particular locality, by stimulating intellectual activity on the part of the members, and promoting good will, so that each meeting will be full of profit to each member in both his head and his heart. Happy is the society that possesses such officers. We have a few such in mind but they are rare, and not always appreciated when they are found.

Third, no doctor will long have an interest in attending the meetings of a medical society who does not himself contribute some thought, or observation, or experience, or study to the society. If he takes part in the work of the society, then he becomes an integral portion of it, otherwise he is a looker-on, a critic of others' thoughts or work. Hence in so far as possible each member should be induced to work in and with the society —become a living portion of its body. In this manner he will have cultivated one of the most important requisites of a doctor's success, viz., the power to talk standing, clearly, interestingly and forcibly.

To a larger degree than most doctors are aware, they are judged in the minds of the laity, by their conversation. If this be such as to commend itself to educated and thinking people a marked advantage is had over a doctor equally well qualified, who cannot express with fluency his views on the subjects he is called on to discuss with patients and outsiders. We emphasize this point because in our observations excellent doctors fail to avail themselves of the advantages of medical societies for such training, and so are at a disadvantage with other doctors possessing less actual knowledge and ability, but more power of forceful, pleasing conversation or speech.

Fourth, it is possible to organize a combined study of a certain subject by all the members of any society, by which should be brought to a common center all the individual knowledge of the members. It is well known that types of given diseases vary under varied conditions, and that these conditions are never identical, in different localities, social industrial, etc., etc., conditions. Is it impossible to interest members of a society in the study of the pecularities of detail in the diseases incident to their field of of observation ?—American Lancet.

---

WE ARE AUTHORIZED by State Health Officer Rutherford to announce that he has no connection whatever, as "associate editor" or otherwise, with any Health Journal in Texas.

